# Rehabilitation or Stagnation? a Six Month Mirror-Image Study Reviewing the Effectiveness of an Inpatient Rehabilitation Unit

**DOI:** 10.1192/bjo.2022.310

**Published:** 2022-06-20

**Authors:** Adisha Kapila, Eromona Whiskey, Mehak Nagpal, Patrick Davey, Rebekah King

**Affiliations:** 1 South London & Maudsley NHS Foundation Trust, London, United Kingdom; 2 Institute of Psychiatry, Psychology & Neuroscience, London, United Kingdom

## Abstract

**Aims:**

The Tony Hillis Unit (THU) is a locked rehabilitation unit for men aged 18–65 years with Treatment Resistant Psychosis, with or without mild personality disorders; drug and alcohol misuse; and challenging behaviour. The multi-disciplinary team including psychiatrists, psychologists, nurses, occupational therapists and specialist pharmacists offer service-users a holistic, personalised and pragmatic management plan to facilitate an improvement in their level of functioning. This service evaluation aimed to review the effectiveness of our intervention as a unit as defined by functional outcome at six months pre- and post- admission.

**Methods:**

A retrospective, mirror-image study design was used to collect data. Data were obtained from South London & Maudsley's Electronic Patient Journey System (ePJS) records. All patients discharged from THU over a two-year period, from May 2019 to May 2021, were considered in the study (n = 25 patients). Two service users died during the evaluation period and were excluded. A further service user was excluded as he had an admission length less than 28 days. Variables recorded included patient demographics and the presence of biopsychosocial interventions at THU including Clozapine initiation, engagement in weekly 1:1 occupational therapy (OT) and 1:1 psychology sessions. The functional status at six months pre-admission and post-discharge was defined by placement type, graded in terms of level of support; 1 = Psychiatric Intensive Care Unit, 2 = Acute ward, 3 = Rehabilitation service/Prison, 4 = Care home, 5 = Supported accommodation and 6 = Independent living. The change in patient acuity pre- and post- THU was compared using Wilcoxon-signed rank test.

**Results:**

23 service users were included in this evaluation. The average admission length was 365 days, and average age at admission was 38 years. The difference in patient acuity before and after THU intervention was statistically significant (P < 0.005), with an overall reduction in level of placement support required. The most common placement prior to admission was an acute ward, compared to a rehabilitation service six months after discharge. 60% of patients (n = 13) were newly initiated or re-titrated on Clozapine during their admission, with a further 4 patients already on Clozapine. 82% of patients engaged with 1:1 weekly OT and 72% engaged with 1:1 weekly psychology sessions.

**Conclusion:**

This study demonstrates the effectiveness of our role as a locked rehabilitation unit. It outlines some of the key biopsychosocial interventions likely contributing to this, including initiation of Clozapine.

